# Postponing parenthood to advanced age

**DOI:** 10.1080/03009734.2016.1201553

**Published:** 2016-07-05

**Authors:** Ulla Waldenström

**Affiliations:** Department of Women’s and Children’s Health, Division of Reproductive Health, Karolinska Institutet, Stockholm, Sweden

**Keywords:** Advanced maternal age, advanced paternal age, delayed childbearing, postponing parenthood

## Abstract

The aim of the Postponing Parenthood project was to investigate several aspects of the delaying of childbearing phenomenon in Sweden and Norway, such as medical risks and parental experiences. Data were retrieved from the Swedish and Norwegian Medical Birth Registers and three different cohorts: the Swedish Young Adult Panel Study, the Norwegian Mother and Child Cohort, and the Swedish Women’s Experiences of Childbirth cohort. Postponing childbirth to age 35 years and later increased the risk of rare but serious pregnancy outcomes, such as stillbirth and very preterm birth. Older first-time parents were slightly more anxious during pregnancy, and childbirth overall was experienced as more difficult, compared with younger age groups. First-time mothers’ satisfaction with life decreased from about age 28 years, both when measured during pregnancy and early parenthood. Delaying parenthood to mid-30 or later was more related to lifestyle than socioeconomic factors, suggesting that much could be done in terms of informing young persons about the limitations of fertility and assisted reproductive techniques, and the risks associated with advanced parental age.

Parental age when having a first child in Sweden and Norway has increased by five years compared with the previous generation (Figure 1), and a similar development has taken place in many other high-income countries (1,2). This development is problematic for several reasons. Many couples may have difficulties in becoming biological parents because of declining fertility, or in having the number of children they wish. Treatment for involuntary childlessness can be an expensive, time-consuming, and draining procedure (3). Childbirth at advanced maternal age is associated with medical interventions and adverse pregnancy outcomes (4). Besides the individual risks, postponing childbirth to advanced age is associated with economic cost for society. Increasing age at first birth may also partly explain the declining birth-rate in some countries (5). In Sweden and Norway, however, this has not yet become a problem, probably due to supportive family policies with generous paid parental leave and public child care, which have made it easier to combine parenthood and professional career.

**Figure 1.**
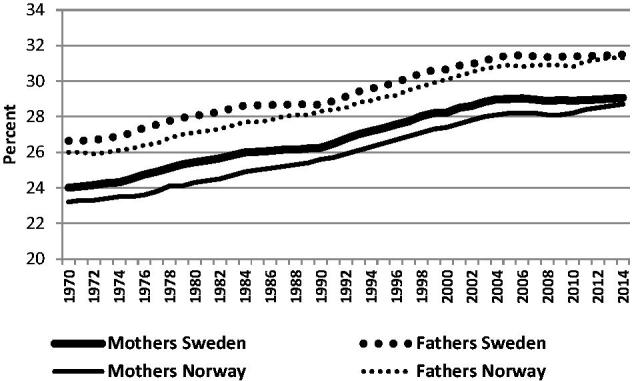
Mean age of first-time mothers and fathers in Sweden and Norway 1970–2014. Source: Statistics Sweden and Statistics Norway.

The *Postponing Parenthood* project aimed at providing a comprehensive picture of the phenomenon by describing different aspects, such as why many women and men have their first child at advanced age, and possible consequences in terms of pregnancy outcomes and parental experiences during pregnancy and the postnatal period. This article presents some of the major findings of the project under the following headlines:

- Why postponing parenthood?
- Risk of adverse pregnancy outcomes
- Experiences of childbirth
- Emotional well-being

The studies were based on data from the Swedish and Norwegian Medical Birth Registers and three different cohorts: the Swedish Young Adult Panel Study (YAPS) (www.suda.su.se/yaps), the Norwegian Mother and Child Cohort (MoBa) (www.fhi.no/den-norske-mor-og-barn-undersokelsen), and the Swedish Women’s Experiences of Childbirth cohort (KUB). Secondary analyses of data from a randomized controlled trial of antenatal education were also used.

There is no consensus on how to define advanced maternal and paternal age in relation to a first pregnancy. In women, 35 years is a commonly used age cut-off in research, but other definitions have also been used, such as the upper quartile of the age distribution. The rationale for the definitions used in *Postponing Parenthood* is given in the publication from the project.

## Why postponing parenthood?

### Family background

We investigated predictors of still being childless at age 32 years by using longitudinal data from YAPS. Women and men answered questions about their family background and attitudes to children at the age of 22, and their answers were linked to information about childlessness obtained from the Swedish Total Population Register 10 years later (6). At that time point, 34% of the 518 women were childless and 46% of the 482 men. Table 1 shows that predictors of being childless were: growing up in a large city, having well-educated parents, no siblings, and not having moved from home at age 22. Other factors associated with childlessness at age 32 were less than positive experiences of one’s own parents, specifically the relationship with the mother, and a negative attitude to children in general.

**Table 1.** Associations between childlessness at age 32 and family background, assessment of own parents, and attitudes to children reported at age 22 years (*n* = 1000).

Questionnaire data at age 22	Odds ratio	95% CI
Family background		
Growing up in a large city	1.5	1.1–2.0
Well-educated mother	1.5	1.1–1.9
Well-educated father	1.4	1.1–1.9
No siblings	1.9	1.1–3.2
Living in parental home	2.2	1.6–3.1
Assessment of own parents		
Mother less than good as a parent	1.8	1.2–2.7
Father less than good as a parent	1.4	1.0–1.9
Less than satisfactory relationship with mother	1.5	1.1–2.1
Attitudes to children		
Not enjoying children	2.7	2.0–3.6
Not assuming that one will have children in the future	2.9	2.1–3.9

Analyses by bivariate logistic regression. Data retrieved from Tables 2 and 3 in Nilsen et al., 2015 (6).

CI: confidence interval.

### Childless persons’ own explanations

**Figure 2.**
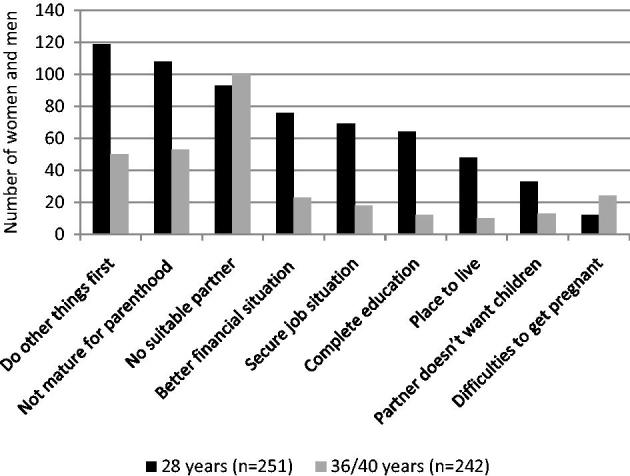
Reasons for remaining childless in women and men at age 28 and 36 + 40 years. More than one reason possible. Source: Data obtained from Schytt et al., 2014 (7).

Cross-sectional data from YAPS were used to investigate reproductive intentions in a subsample of women and men at age 28, 32, 36, and 40 years who were still childless, in total 365 women and 356 men (7). Reasons for remaining childless in the youngest (28 years) and oldest groups (including 36- and 40-year-olds) are presented in Figure 2. Not yet being prepared for parenthood (wanting to do other things first, and not feeling mature enough to be a parent) followed by not having met a suitable partner were the most commonly given reasons in the youngest group, and not having met a suitable partner in the oldest group. These factors were more important than socioeconomic factors, such as work, finances, and dwelling.

### Characteristics of women and men expecting their first child

**Figure 3.**
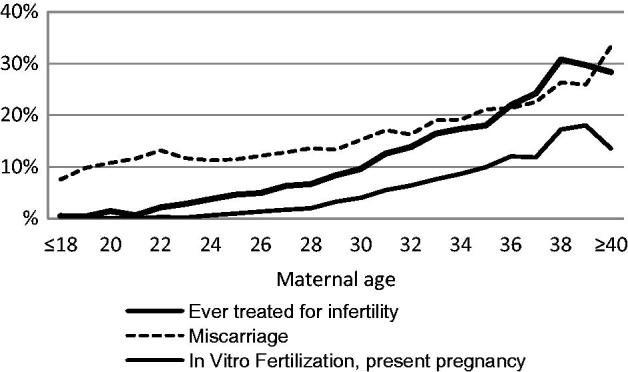
Rates of nulliparous women who have had a miscarriage or been treated for infertility, related to maternal age (*n* = 41,236 women pregnant with their first child). Source: Figure based on a previously published figure by Nilsen et al., 2012 (8).

Studies based on data from MoBa investigated characteristics of 41,236 women (8) and 14,832 men (9) who were expecting their first child. The participants completed questionnaires in gestational week 17–18. Associations between sociodemographic factors, reproductive background, lifestyle, and health problems, and the risk of being of advanced age (defined as 33–37 years in women, and 35–39 years in men) or very advanced age (38 years and older in women, and 40 years and older in men) compared with being younger (25–32 years in women, 25–34 years in men) were investigated. The older groups were more educated and had a higher income than the comparison group, but the oldest group was not entirely homogeneous. Very advanced maternal age was also associated with low level of education, being single, unemployed, having an unplanned pregnancy and an unsatisfactory relationship with the partner. The oldest men had more health problems and more risky health behaviour, such as overweight, obesity, smoking, and alcohol intake. Also, advanced maternal age was strongly associated with problems related to fecundity, such as history of miscarriage and *in-vitro* fertilization (Figure 3).

### Comments

By exploring reasons for postponing childbirth from different perspectives, a more complex pattern emerged than would have been the case if we had only asked young people about their subjective opinions. Most studies of women’s and men’s attitudes to parenthood and opinions about the appropriate time of having a first child have focused on the young, and specifically on students. These studies show that most university students in countries like Sweden (10–13), Finland (14), UK (15), Canada (16), USA (17), and Australia (18) wish to have children, and usually more than one child. Lifestyle factors such as not feeling sufficiently mature for parenthood and not living in a stable relationship are often rated as the most important reasons for waiting (10,13). Our population-based sample of older participants supports these findings (7), but the other studies in *Postponing Parenthood* add additional explanations.

The longitudinal study indicated that experiences during childhood may have long-lasting effects on the timing of parenthood, and such factors are not revealed when adults think about reasons why they are still childless. Many of the associations found in Table 1 have also been reported by others (19–21), although a negative experience of one’s own parents has also been associated with childbirth at young age (22).

The description of background characteristics of expecting first-time parents in MoBa pointed at another factor that may be sensitive to disclose when direct questions are asked about reasons for delaying childbirth, and that is problems getting pregnant. Figure 3 illustrates that older women may well have tried to become pregnant several years earlier without success. This may be a reflection of low fertility awareness, which was found in the oldest participants in YAPS (36 + 40 years), of whom one-third of the women and half of the men thought they could continue to postpone the first pregnancy (7). Similar trends have been reported in Swedish university students, half of whom planned to have children after age 35 years (10). An interview study of highly educated and still childless women and men who were at the beginning of their professional careers described fertility as an imperceptible and retrievable capacity, and postponed parenthood as a rational adaptation to changes in society (3).

At age 28 years, around 35% listed completion of education and a better financial situation as important reasons for postponing parenthood (Figure 2), but these factors were less important in the oldest group. Two principal explanations of the postponement of childbirth phenomenon are women’s increased participation in the labour market, including longer education and career engagement, and couples’ inclination to schedule the first child to a point in time when family income is high (2,23–25). Our findings suggest that these are relevant explanations of why a first pregnancy occurs around the age of 30, but less important at age 35 years and older.

Naming our project *Postponing Parenthood* was based on the development of parental age over the last three decades in Sweden and Norway: that parenthood was postponed or delayed compared with the previous generation. Our studies elucidate that the question ‘Why postponing childbirth?’ needs further specification, since the reasons may vary depending on what age the postponing refers to. The findings suggest that experiences from family of origin, lifestyle factors such as difficulties finding a suitable partner, and limited fertility awareness are major explanations for postponing parenthood to an age beyond 30 years, or even beyond 35 years.

## Risk of adverse pregnancy outcomes

### Advanced maternal age, smoking, and overweight

Our first publication on medical risks investigated associations between advanced maternal age and some of the most serious outcomes of pregnancy, and compared risks related to advanced maternal age with those related to smoking and being overweight or obese (26). We obtained data from the Swedish and Norwegian Medical Birth Registers on all nulliparous women aged 25 years and older with singleton pregnancies at 22 weeks of gestation or greater who gave birth from 1990 to 2010, in total 955,804 pregnancies. In each national sample, adjusted odds ratios (ORs) of very preterm birth, moderately preterm birth, small for gestational age, low Apgar score, fetal death, and neonatal death in women aged 30–34 years, 35–39 years, and 40 years or older were compared with those of women aged 25–29 years.

**Figure 4.**
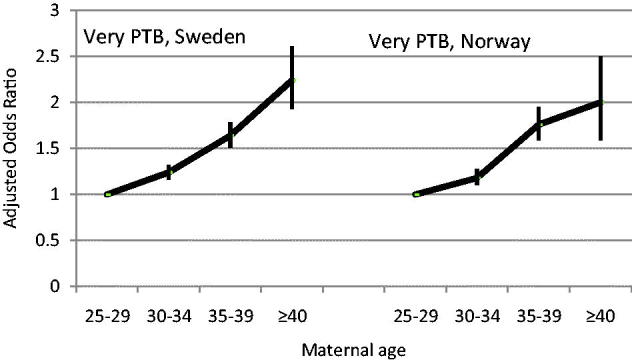
Risk of very preterm birth (22–31 gestational weeks) (PTB) in relation to advanced maternal age in primiparous women in Sweden and Norway. Adjusted odds ratios and 95% confidence intervals (CI). Sweden: *n* = 644,184; Norway: *n* = 311,620. Logistic regression analyses adjusted for year of birth, civil status, country of birth, smoking, BMI, chronic hypertension, and diabetes. Source: Figure based on data obtained from Table 1 in Waldenström et al., 2014 (26).

The adjusted OR of all outcomes increased by maternal age in a similar way in Sweden and Norway. As one example, Figure 4 illustrates the risk of very preterm birth, which increased even in the 30–34-year-old group, but more obviously at age 34–39 years (Sweden: adjusted OR 1.64, 95% CI 1.51–1.78; Norway: adjusted OR 1.76, 95% CI 1.59–1.95).

**Figure 5.**
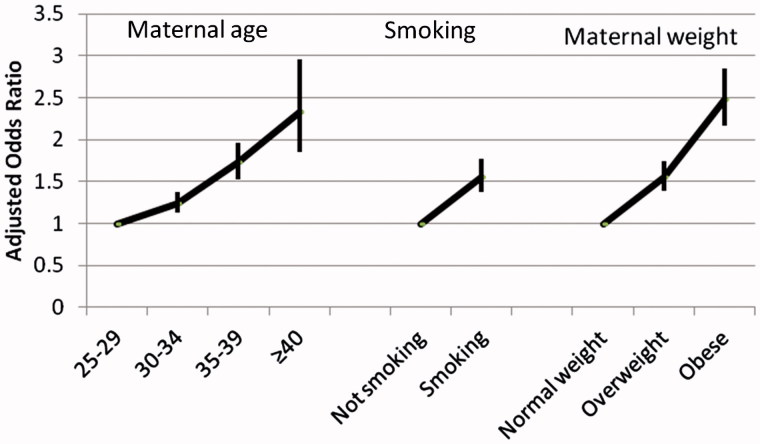
Risk of stillbirth in relation to advanced maternal age, smoking in early pregnancy, and being overweight or obese. Adjusted odds ratios and 95% confidence intervals (*n* = 644,184 primiparous women). Logistic regression model including maternal age, smoking, body mass index, and the following potentially confounding factors not shown: year of birth, civil status, country of birth, chronic hypertension, and diabetes. Source: Figure based on data obtained from Table 2 in Waldenström et al., 2014 (26).

Figure 5 shows how the risk of stillbirth increases by the maternal age, smoking, and overweight/obesity in the Swedish sample. The three lifestyle factors were independent risk factors for stillbirth.

The study concluded that the absolute risk for each outcome was small for the individual woman, but may be significant for society as a result of the large number of women who give birth after the age of 30 years.

### Risk of stillbirth by maternal age and parity

Most research on adverse pregnancy outcomes in relation to advanced maternal age have either investigated nulliparous women only, nulliparous women and a merged group of all parous women, or treated parity as a confounder in the statistical analyses. We therefore continued to focus more in-depth on the most serious outcomes, such as stillbirth (27) and preterm birth (28), and investigated associations with advanced maternal age in first, second, third, and fourth birth or more.

Women aged 25 years and older with singleton pregnancies at 28 weeks of gestation and later who gave birth in Sweden from 1990 to 2011 were included in the study of stillbirth, in total 1,804,442 pregnancies. In each parity group, the risks of stillbirth at age 30–34 years, 35–39 years, and 40 years and older, compared with age 25–29 years, were investigated by logistic regression analyses adjusted for sociodemographic factors, smoking, body mass index, history of stillbirth, and inter-delivery interval.

Stillbirth risk increased by maternal age in first births. Compared with age 25–29 years, this increase was approximately 25% at 30–34 years and doubled at age 35 years. In second, third, and fourth birth or more, stillbirth risk increased with maternal age in women with a low and middle level of education, but not in women with high education (Table 2).

**Table 2.** Stillbirth in relation to maternal age in first, second, third, and fourth or more births stratified by level of education.

Maternal age	Education: low or medium (*n* = 993,937)			Education: high (*n* = 771,443)		
Year	%	*n*	Adjusted OR 95% CI	%	*n*	Adjusted OR 95% CI
1st births						
25–29	0.34	646	Ref = 1	0.22	343	Ref = 1
30–34	0.44	395	1.28 (1.13–1.45)	0.27	383	1.23 (1.07–1.43)
35–39	0.57	159	1.56 (1.31–1.86)	0.44	186	1.86 (1.55–2.22)
≥40	0.71	33	1.86 (1.31–2.65)	0.67	49	2.72 (2.01–3.69)
2nd births						
25–29	0.24	478	Ref = 1	0.23	169	Ref = 1
30–34	0.30	408	1.23 (1.08–1.41)	0.21	308	0.94 (0.78–1.14)
35–39	0.37	171	1.40 (1.17–1.68)	0.28	179	1.18 (0.95–1.47)
≥40	0.55	38	1.98 (1.41–2.76)	0.28	29	1.12 (0.75–1.68)
3rd births						
25–29	0.27	180	Ref = 1	0.23	23	Ref = 1
30–34	0.34	267	1.25 (1.03–1.51)	0.25	109	1.18 (0.75–1.86)
35–39	0.50	192	1.87 (1.51–2.31)	0.25	99	1.23 (0.77–1.95)
≥40	0.65	46	2.36 (1.68–3.30)	0.29	22	1.41 (0.77–2.57)
4th births or more						
25–29	0.39	73	Ref = 1	0.34	4	Ref = 1
30–34	0.47	188	1.25 (0.95–1.64)	0.34	26	1.08 (0.38.3.13)
35–39	0.51	168	1.34 (1.01–1.79)	0.35	44	1.12 (0.39–3.16)
≥40	0.66	70	1.72 (1.22–2.43)	0.42	22	1.29 (0.43–3.86)

Logistic regression analyses adjusted for year of birth, family situation (living with baby’s father compared with not), country of birth (Sweden compared with not Sweden), smoking in early pregnancy, maternal height, BMI, history of stillbirth in previous pregnancy, and number of years from previous to present pregnancy. Data obtained from Table 2 in Waldenström et al., 2015 (27).

We concluded that the findings in the high-education groups were least biased by unmeasured confounding, and therefore the most valid in this context in which focus was on possible effects of biological ageing. The article concluded: ‘Advanced maternal age is an independent risk factor for stillbirth in nulliparous women. This age-related risk is reduced or eliminated in parous women, possibly as a result of physiologic adaptations during the first pregnancy’ (27).

### Comments

Placental pathology is a major cause of stillbirth, followed by infection, cord complication, maternal medical disorders, congenital anomalies, and intrapartum events, but in about 30% of cases no explanation is found (29,30). There is considerable indirect evidence that utero-placental blood flow decreases by maternal age; and sclerotic lesions in the myometrial arteries could be one cause of underperfusion (31). This conclusion was based on analyses of uteruses from 62 non-pregnant women who came to autopsy as a result of accidental death, and the proportion of such lesions increased from 11% at age 17–19 years to 83% after age 39 years. These age-related vascular changes did not differ by parity. This may explain the overall effect of advanced maternal age on stillbirth risk, but not our finding that the age-related risk was restricted to first births.

Having experienced a first pregnancy may possibly improve the physiologic conditions for the second birth in a way that affects the fetal environment. One hypothesis could be that some of the effects of the hemodynamic changes that occur during the first pregnancy persist during the subsequent pregnancy and facilitate the blood transfusion to the next fetus (32–36). If structural changes during the first pregnancy have a positive effect of placental perfusion during the second pregnancy, this could reduce the negative effects of the age-related vascular lesions. The positive effect may be limited in time and possibly negated by further effects of maternal ageing on the vascular bed, and this could explain the observed trend in our study of an increased risk of stillbirth by maternal age in third births.

These findings illustrate that ageing in younger women does not only affect the human egg but also other vital organs and structures. In our subsequent work on associations between advanced maternal age and risks of preterm birth we speculate that the age-related decline in progesterone may also be important (28).

Public awareness in Sweden is high in relation to negative effects of smoking during pregnancy, with smoking habits being addressed at the first antenatal visit (37). The negative effects of overweight/obesity and advanced maternal age have still not gained the same public attention, probably because research evidence showing these factors’ associations with negative pregnancy outcomes is more recent (4,38,39). Our studies add to the increasing body of research showing that advanced maternal age should be regarded as a modifiable lifestyle factor that could affect pregnancy outcomes.

## Experiences of childbirth

The focus of one of the first studies in the *Postponing Parenthood* project was women’s experience of childbirth in relation to maternal age (40). In total, 1302 nulliparous women from the Swedish KUB cohort were included. The women were recruited at their first booking visit, at 593 antenatal clinics in Sweden (97% of all clinics), during three 1-week periods evenly spread over one year in 1999 to 2000. Questionnaires were posted in the second trimester and two months after the birth.

**Figure 6.**
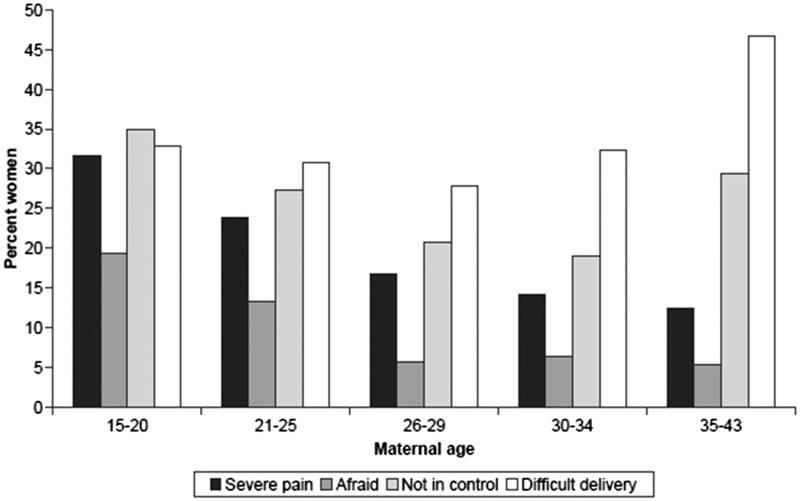
Postnatal assessment of childbirth by maternal age (*n* = 1184 primiparous women). Source: Figure previously published by Zasloff et al. 2007 (40).

Figure 6 shows that the oldest women reported the most negative overall assessment, with 45% saying the delivery was difficult, whereas the younger women reported more pain and having been afraid. Lack of control was most commonly reported by the youngest, followed by the oldest women.

The rates of elective and emergency caesarean section increased continuously by maternal age. Only 57% of the oldest women had a normal vaginal delivery compared with 77% in the youngest group. In addition, 7% of the newborns in the oldest group were transferred to the neonatal clinic after the birth, compared with 1.6% in the youngest.

**Figure 7.**
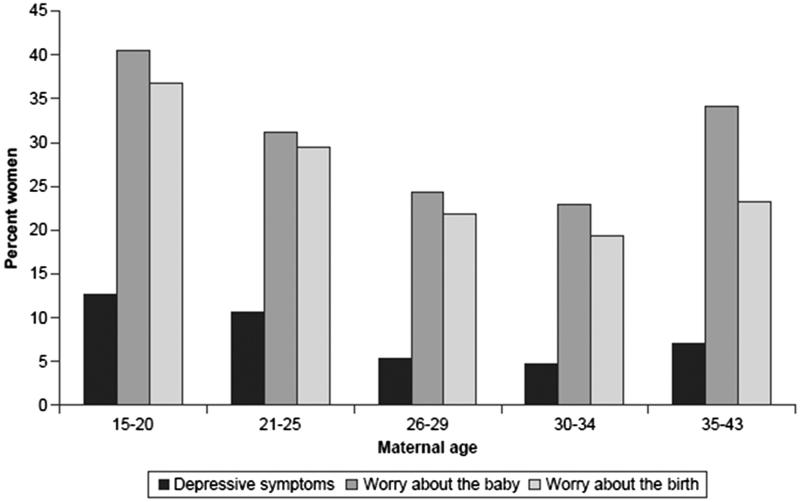
Depressive symptoms and worries during pregnancy by maternal age (*n* = 1304 primiparous women). Source: Figure previously published by Zasloff et al. 2007, page 1331 (40).

During pregnancy, the distributions of depressive symptoms measured by the Edinburgh Postnatal Depression Scale (41) and worries about the baby and upcoming birth measured by the Cambridge Worry Scale (42) were U-shaped, with the highest rates in the youngest age group, followed by a continuous decline and a small increase at age 35 years and older (Figure 7).

The younger women in the study were more exposed to problems, such as low level of education, and being single and unemployed, factors that may have contributed to depressive symptoms and worries during pregnancy and experiences of fear, pain, and lack of control during labour. The oldest women may have suffered from the biological disadvantage of high maternal age, with its association with complicated delivery and adverse outcomes. In 2014, the caesarean section rate in Sweden was 35% in nulliparous women aged 35 years and older, compared with 13% in women younger than 25 years. The corresponding figures for instrumental vaginal delivery were 21% versus 10%.

The KUB study was followed by a study based on data from the Norwegian MoBa cohort in which the childbirth experiences of 30,065 nulliparous women of advanced age (defined as ≥32 years) were compared with a reference group of 25–31 years (43). Women in the study were recruited in the second trimester during the period 1999 to 2008. Three questionnaires were completed: around gestational weeks 17 and 30, and 6 months after the birth.

Women of advanced age were slightly more worried about the upcoming birth than were the reference group (adjusted OR 1.13; 95% CI 1.06–1.21), a finding consistent with the proportions presented in Figure 6 from the KUB study (see ‘Worry about the birth’ in the three oldest age categories). Six months after the birth the older MoBa women had a slightly higher risk of having experienced childbirth as ‘worse than expected’ (adjusted OR 1.09; 95% CI 1.02–1.16). Comparisons with KUB findings are difficult since the women were asked to rate their experience of childbirth in relation to their expectations, and not on a scale ranging from ‘very easy’ to ‘very difficult’ as in the KUB study.

Experiences of childbirth were also investigated in first-time fathers by conducting secondary analyses of data from a randomized controlled trial of antenatal education. In total, 777 first-time fathers completed questionnaires when their partners were in mid-pregnancy and three months after the birth (44). They were divided into three age groups: young age (≤27 years), average age (28–33 years), and advanced age (≥34 years). During pregnancy, mixed or negative feelings about the upcoming birth were more prevalent in the oldest men (29%), compared with men of average (26%) or young (18%) age (*P* < 0.01). Also, the older men had higher scores of childbirth fear, measured by the Wijma Delivery Expectancy Questionnaire (45) (mean 43.3, 42.9, and 38.7, respectively; *P* < 0.01). After the birth, a larger proportion of the oldest men reported that their partner’s labour and birth were difficult, compared with the men of average and young age (43%, 41%, and 32% respectively; *P* < 0.05), and older men’s overall experience of childbirth was less than positive (30%, 36%, and 43% respectively; *P* < 0.05).

### Comments

The KUB and MoBa studies illustrate that conclusions about possible consequences of delaying childbirth on women’s feelings during pregnancy and experiences of childbirth are dependent on the definition of ‘advanced age’, the definition of the comparison group, and how women’s overall childbirth experience is measured. The two studies have their strengths and limitations. The KUB study was smaller than the MoBa study but more representative of the total population because of a higher rate of women who consented to participate and higher response rates to the questionnaires. The MoBa sample was more skewed, compared with all nulliparous women of the same age who gave birth in Norway during the same time period (under-represented characteristics: smoking, single status, IVF pregnancy, caesarean delivery, preterm birth, infant transfer to neonatal unit). In contrast to the KUB study, the MoBa study analysed data by logistic regression analyses, which were adjusted for potentially confounding factors, such as education, native language, civil status, and smoking. In spite of these differences the two studies suggest that advanced maternal age has a slightly negative effect on emotional well-being during pregnancy and the overall experience of childbirth. The secondary analyses of data on first-time fathers suggest that advanced paternal age has similar effects.

## Emotional well-being

Another study based on MoBa data investigated if advanced maternal age at first birth increased the risk of psychological distress during pregnancy at 17 and 30 weeks of gestation and at 6 and 18 months after the birth (46). A total of 19,291 nulliparous women were included. Psychological distress was measured by a short version of the Hopkins Symptom Checklist (SCL-5) (47), which is a five-item scale including two dimensions: depressiveness (three items) and anxiousness (two items), and distress was defined as SCL-5 scores 1.75 or higher.

**Figure 8.**
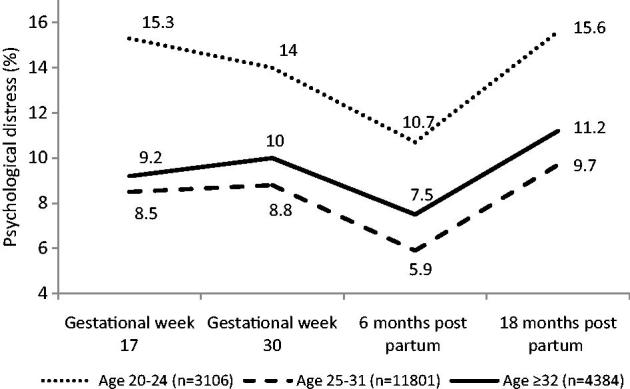
Psychological distress (SCL-5 ≥ 1.75) in primiparous women by maternal age, in weeks 17 and 30 of gestation and 6 and 18 months after birth (*n* = 19,291 primiparous women). Source: Figure previously published by Aasheim et al., 2012 (46).

Figure 8 shows that psychological distress was most prevalent in the youngest women (20–24 years), and slightly more common in the oldest group (≥32 years) compared with the 25–31-year-olds. The figure also shows that the lowest rates were found at 6 months after the birth, regardless of maternal age.

When analysing the risk of psychological distress longitudinally by generalized estimation equation (GEE) from pregnancy to 18 months after birth, and adjusting for potentially confounding factors (time point, sociodemographics, operative delivery, and infant outcomes), the risk of psychological distress in the oldest group was only marginally increased compared with the reference group aged 25–31 years: adjusted OR 1.14 (95% CI 1.04–1.25).

In the last study based on MoBa data, satisfaction with life was investigated in 18,565 nulliparous women (48). This study also included a follow-up at 3 years after the birth. Satisfaction with life was measured by the five-item version of the Satisfaction With Life Scale (SWLS) (49,50), including the following statements: ‘My life is largely what I wanted it to be’, ‘My life is very good’, ‘I am satisfied with my life’, ‘I have achieved so far what is important to me in my life’, and ‘If I could start all over again, there is very little I would do differently’. The responses were given on seven-point Likert scales ranging from ‘Totally disagree’ (=1) to ‘Totally agree’ (=7).

**Figure 9.**
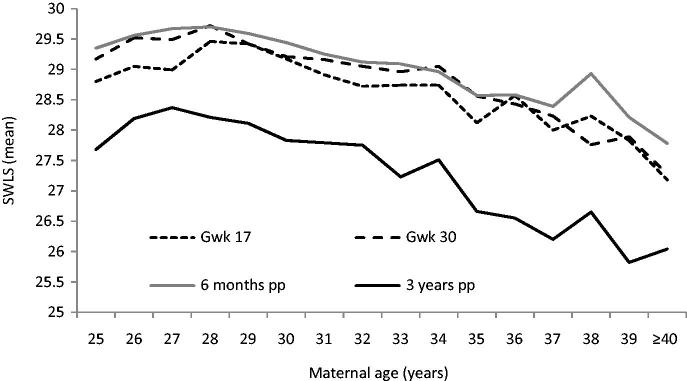
Satisfaction with life (SWLS, mean score) in gestational weeks 17 and 30 and at 6 months and 3 years after birth in relation to maternal age (*n* = 5891 primiparous women). Source: Figure previously published by Aasheim et al., 2014 (48).

Figure 9 shows that mean SWLS scores decreased from around age 28 years to age 40 and older, when measured in gestational weeks 17 and 30, and at 6 months and 3 years after the birth.

Satisfaction with life was higher in this sample of childbearing women than in a sample of 1183 women drawn from the total Norwegian population of women at the same age, and who were investigated with the same SWLS. Three years after birth, the scores of the new mothers had decreased to about the same as in the population sample. In contrast to the new mothers’ assessments, satisfaction with life increased by age in the population-based sample.

When analysing satisfaction with life longitudinally by GEE from gestational week 17 to 3 years after birth, and adjusting for potentially confounding factors (time point and sociodemographics), satisfaction with life was slightly reduced in women of advanced age (here defined as 32–37 years) compared with a reference group of 25–31 years (mean difference –0.7; 95% CI –0.83 to –0.58). In women of very advanced age (defined as 38 years and older) the difference was more pronounced (mean difference –1.32; 95% CI –1.65 to –0.99).

### Comments

Biological ageing may be an important explanation of these findings, considering that age-related physical health problems such as fatigue and sleeping problems (8), obstetric complications (4), negative birth experiences (40,43), and infant health problems (51,52) are more frequent in nulliparae of advanced age. Older first-time mothers may also be less prepared for the unpredictable life of parenthood after having been used to a higher degree of control during many years. Still another explanation could be related to the selection of women who had their first child at an advanced age, and the possibility that important confounding factors were not taken into account.

## Conclusions

Many of the outcomes of the *Postponing Parenthood* project are confirmations of previously reported findings, such as reasons for delaying childbirth and risks of adverse pregnancy outcomes. However, the project also adds to or deepens our knowledge about the phenomenon, at least in high-income countries like Sweden and Norway.

1. Reasons for delaying parenthood to advanced maternal age defined as around 35 years and older are more related to lifestyle factors than to socioeconomic factors, such as completion of one’s education, financial security, and place to live. Possibly, lifestyle factors could more easily be affected by information about the risks associated with advanced maternal age. Our findings regarding fertility awareness suggest that much can be done in terms of informing young persons about fertility, the age-related limitations of assisted reproductive techniques, and medical risks associated with pregnancy at advanced age.
2. Advanced maternal age defined as 35 years and older in nulliparous women may increase the risk of very preterm birth and fetal death in similar ways as smoking and overweight/obesity.
3. By studying the risk of adverse pregnancy outcomes by advanced maternal age in different parity groups, new hypotheses about age-related physiological mechanisms during pregnancy have been presented. Work in progress investigates associations between advanced maternal age and preterm birth, and advanced maternal age and obstetric anal sphincter injury, in first, second, and third births.
4. Anxiety during pregnancy and a negative overall experience of childbirth is slightly more common in first-time parents of advanced age. Higher rates of difficult deliveries, with labour ending with an emergency caesarean section or instrumental vaginal delivery, contribute to these findings.
5. A common view is that postponing parenthood to advanced age may be beneficial because of a higher degree of socioeconomic stability and parental maturity. However, this statement is challenged by our finding that first-time mothers’ satisfaction with life decreased by age, suggesting that becoming a parent later in life may be more difficult than expected.
